# Early detection of *Mycobacterium avium* subsp. *paratuberculosis* infection in cattle with multiplex-bead based immunoassays

**DOI:** 10.1371/journal.pone.0189783

**Published:** 2017-12-19

**Authors:** Lingling Li, Bettina Wagner, Heather Freer, Megan Schilling, John P. Bannantine, Joseph J. Campo, Robab Katani, Yrjo T. Grohn, Jessica Radzio-Basu, Vivek Kapur

**Affiliations:** 1 Department of Animal Science, The Pennsylvania State University, University Park, PA, United States of America; 2 Huck Institutes of Life Sciences, The Pennsylvania State University, University Park, PA, United States of America; 3 Department of Population Medicine and Diagnostic Sciences, Cornell University, Ithaca, NY, United States of America; 4 National Animal Disease Center USDA-ARS, Ames, IA, United States of America; 5 Antigen Discovery, Inc., Irvine, CA, United States of America; Indian Institute of Technology Delhi, INDIA

## Abstract

Johne’s Disease (JD), caused by *Mycobacterium avium* subspecies *paratuberculosis* (MAP), results in significant economic loss to livestock production. The early detection of MAP infection in animals with extant serological assays has remained challenging due to the low sensitivity of commercially available ELISA tests, a fact that has hampered the development of effective JD control programs. Our recent protein microarray-based studies identified several promising candidate antigens that are immunogenic during different stages of MAP infection. To evaluate these antigens for use in diagnostic assays and reliably identify animals with MAP infection, a multiplex (Luminex^®^) assay was developed using color-coded flourescent beads coupled to 6 MAP recombinant proteins and applied to screen 180 serum and 90 milk samples from cows at different stages of MAP infection including negative (NL), fecal test positive/ELISA negative (F+E-), and fecal positive/ELISA positive (F+E+). The results show that while serum antibody reactivities to each of the 6 antigens were highest in F+E+ group, antibody reactivity to three of the six antigens were identified in the F+E- group, suggesting that these three antigens are expressed and provoke antibody responses during the early infection stages with MAP. Further, antibodies against all six antigens were elevated in milk samples from both the F+E- and F+E+ groups in comparison to the NL group (*p*<0.01). Taken together, the results of our investigation suggest that multiplex bead-based assays are able to reliably identify MAP infection, even during early stages when antibody responses in animals are undetectable with widely used commercial ELISA tests.

## Introduction

Johne’s disease (JD) is a chronic granulomatous intestinal inflammatory disease that results from infection with *Mycobacterium avium* subspecies *paratuberculosis* (MAP) [1]. Although animals are infected early in life through ingestion of bacilli via the fecal-oral route or from colostrum, JD takes several years to manifest [2,3]. During this extremely long sub-clinical phase, infected animals are continuously or intermittently shedding the pathogen into the environment and spreading the disease. JD is recognized as a serious animal health problem in domesticated ruminants including dairy and beef cattle, sheep, and goats, resulting in more than $200 million in annual losses to the US dairy industry with additional losses incurred in other species [4]. The current diagnosis methods of MAP infection including fecal tests and serological immunoassays (ELISA) have been limited in detection of infected from non-infected animals during early infection because it is very difficult to reliably identify infected animals that are intermittently shedding with fecal tests and currently available ELISA assays have low sensitivity in detecting animals with subclinical infection, and only about one third of MAP-infected cows are detected by current ELISA assays in longitudinal studies [5,6].

Current ELISA assays use relatively crude cellular extracts that share antigens with other common mycobacteria and need cumbersome pre-absorption steps in order to ensure specificity [7]. However, this also results in a considerable decrease in analytical and diagnostic sensitivity [8], highlighting the need for more sensitive, high-throughput screening assays to identify MAP-infected animals during the early, subclinical phase. Since the first complete MAP genome sequence was published [9], many studies with recombinant MAP proteins have been conducted to identify potential candidates for use as diagnostic antigens that could distinguish animals with mild or early MAP infection from those uninfected [10–16]. We recently screened a set of well-characterized serum samples using a whole proteome microarray from *Mycobacterium tuberculosis* (MTB), and several promising candidate antigens were identified from these studies as immunogenic during MAP infection [17]. These antigens need to be further evaluated for the development of a high-throughput, diagnostic immunoassay.

One commonly used high-throughput screen technique is fluorescent bead-based multiplex immunoassay that involves 100 distinctly color-coated bead sets created by the use of two fluorescent dyes (internal dye and reporter dye) at distinct ratios (e.g. Luminex^®^, http://www.luminexcorp.com/). Each bead set can be coated with an antigen specific to a particular assay, allowing the capture and detection of a specific analyte from a given sample [18]. For example, a recombinant MAP antigen can be coupled to a bead with one distinct internal dye and is then recognized by a MAP antigen-specific antibody in a sample. This specific antibody is bound by a secondary antibody that is attached to a fluorescent reporter dye. Within the Luminex analyzer, lasers excite the internal dyes that identify the distinct bead color corresponding to one MAP antigen, and the reporter dye identifying the amount of MAP-specific antibodies captured during the assay. Multiple beads with different MAP antigens and different bead color codes can be combined in one assay run. Multiple readings are made on each bead set and result in an individual fluorescent signal for each bead assay. In this way, the technology allows rapid and accurate analysis of up to 100 unique assays within a single sample (multiplexing) [18]. Such multiplex immunoassays have been successfully applied to quantify antibodies to pathogens such as *Borrelia burgdorferi*, *Chlamydia trachomatis*, *Streptococcus pneumoniae*, *Haemophilus influenza*, *Moraxella catarrhalis*, and *equine herpesvirus* in human and animal serum samples [19–22].

The aim of this study was to evaluate candidate antigens that can be used to develop a bead-based multiplex immunoassay which reliably identifies diagnostic markers in both serum and milk samples from MAP infected animals. To our knowledge, no bead-based multiplex assay has yet been developed for detection of MAP infection. Here, we describe the development of a multiplex immunoassay for simultaneous detection of antibodies specific to six candidate recombinant MAP proteins. Five of these proteins (MAP1272c, MAP1569, MAP2609, MAP2942c and MAP1201c+2942c fusion protein) were selected because they displayed the highest levels of sensitivity and specificity in our previous protein array studies [17]. Additionally, MAP2121c was selected based on previous studies that showed significant reactivity to samples from infected animals in previous ELISA studies [10,23] although it was not shown in the MTB array due to the absence of an ortholog in MTB [17]. The results show that multiplex bead-based assays reliably identify cows with MAP infection using both serum and milk samples, even during early stages of infection in animals that were fecal test positive but negative based on widely used commercial ELISAs.

## Materials and methods

### Bovine serum and milk samples

All serum and milk samples were collected as part of the Johne’s Disease Integrated Program (JDIP, http://mycobacterialdiseases.org) diagnostic standards sample collection project and have been previously assayed for fecal and ELISA, as described [17]. Animal use protocols were approved by the Pennsylvania State University ISCUC under numbers 34626 and 43309. In brief, the serum and milk samples used in these studies were collected from cows housed in 13 dairy farms from 4 states: California, Georgia, Minnesota, and Pennsylvania. The herd size ranged from 66 to 1,400, and prevalence of JD ranged from 0 to 53.30% based on serum ELISA tests conducted prior to sample collection. All herds were negative for bovine TB. Each cow was tested for level of MAP shedding in feces as well as serological reactivity. MAP shedding was determined by fecal culture using Herrold's solid medium (HEYM) and two different liquid culture medium systems, BACTEC MGIT and Trek (Becton, Dickinson and Company, Franklin Lakes, NJ); all fecal cultures were confirmed by acid fast staining and PCR tests. Fecal qPCR assays were performed for each animal with the LT TaqMan (ThermoFisher, Waltham, MA) and Tetracore (Tetracore, Rockville, MD) assays. Serum and milk ELISA tests were performed using both the IDEXX kit (IDEXX Laboratories, Inc., ME) and the ParaChek (ThermoFisher, Waltham, MA) according to the manufacturers’ instructions. Samples were selected from 180 cows that were stratified into 3 groups as listed in the table: both fecal and ELISA tests negative, and collected from the herds with previously observed JD prevalence of 0% (NL, n = 60); fecal tests positive and ELISA test negative (F+E-, n = 60); and both fecal and serological tests positive (F+E+, n = 60). Serum samples from all 180 cows and milk samples from 90 out of 180 cows (n = 30 per group) were tested in this study. All data are accessible via https://scholarsphere.psu.edu/concern/generic_works/vt435gf14r.

### Preparation of recombinant proteins

The 6 recombinant MAP proteins selected in this study were expressed as maltose binding protein (MBP) fusion proteins because previous studies demonstrated higher yields as compared to six-His tag clones [24]. The full-length coding sequences for 5 of the 6 genes were amplified from MAP K-10 genomic DNA with 5’ primer containing an *Xba*I and 3’ primer a *Hind* III restriction site and cloned into the pMAL-c5 translational fusion expression vector (New England Biolabs, Beverly, MA, USA). The MAP1201c + 2942c was chemically synthesized, amplified and cloned in a manner similar to the other 5 genes. The vector and amplification products were each digested with *Xba*I and *Hind*III, followed by overnight ligation at 4°C. The products were transformed into *E*. *coli* DH5α and selected on LB agar plates containing 0.10 mg/ml ampicillin. Drug-resistant colonies were screened by PCR and plasmid DNA was sequenced to confirm the presence of the correct insert in each clone [24]. These MBP-tagged recombinant proteins were expressed by induction of 1.0-liter LB broth cultures with 0.3 mM isopropyl-β-d-thiogalactopyranoside (Sigma Chemical Company, St. Louis, MO) for 2.5 h with shaking at 37°C. *E*. *coli* cells were harvested by centrifugation at 4,000 × *g*, re-suspended and subjected to a freeze-thaw cycle at −20°C and sonication. The resulting extracts were purified by affinity chromatography with an amylose resin as per the manufacturer’s instructions (New England Biolabs). Purified protein yields are determined from eluted fractions with a NanoDrop spectrophotometer set at 280 nm. The most concentrated fractions were pooled and dialyzed with three exchanges of PBS at 4°C. Purified protein aliquots were stored at −20°C after protein yield was reassessed by a modified Lowry assay using bovine serum albumin (BSA) as the standard. Each recombinant protein was further evaluated by using GelCode blue (Pierce Biotechnology Inc., Rockford, IL)-stained SDS-PAGE gels to assess purity and expected sizes [24].

### Coupling of recombinant MAP proteins to fluorescent beads

A total of 100 μg of each purified recombinant MAP protein was coupled to fluorescent beads (Luminex, Austin, TX) at room temperature according to the manufacturer’s instructions. MAP1272c was coupled to bead 33, MAP1569 to 34, MAP2121c to 35, MAP2942c to 36, MAP2609 to 37, and MAP1201c+2942c to 38. All centrifugation steps were performed at 14,000 x g for 4 minutes (min). In brief, the beads were resuspended by vortexing and sonication for 20 seconds. For activation, 5x10^6^ beads were washed once in deionized H_2_O. Beads were resuspended in 80 μl of 100 mM sodium phosphate buffer, pH 6.2 and 10 μl of Sulfo-NHS (50 mg/ml,) and 10 μl 1-ethyl-3-[3-dimethylaminopropyl] carbodiimide hydrochloride (EDC, 50 mg/ml, both from Pierce Biotechnology Inc., Rockford, IL) were added and incubated for 20 min. The beads were then washed twice with 50 mM 2-[N-morpholino] ethanesulfonic acid pH 5.0 (MES) and resuspended in MES solution. These activated beads were used for MAP antigen coupling using 100 μg of each antigen. The coupling of the MAP antigens was performed for three hours with rotation. After coupling, the beads were resuspended in blocking buffer (PBS with 1% (w/v) BSA and 0.05% (w/v) sodium azide) and incubated for 30 min. The beads were washed three time in PBS with 0.1% (w/v) BSA, 0.02% (v/v) Tween 20 and 0.05% (w/v) sodium azide (PBS-T), counted and stored in the dark at 2–8°C.

### Luminex multiplex assay

Beads coupled with MAP antigens were sonicated, mixed and diluted in blocking buffer to a final concentration of 1 x 10^5^ beads/ml each. For the assay, 5 x 10^3^ beads/antigen were used per microtiter well. Serum samples were diluted 1:400 and milk samples were diluted 1:2 in blocking buffer. In addition to the samples, a set of three previously determined (NL, F+E- and F+E+) serum and milk samples were run on each plate together with a buffer control. These standard and blank samples were used as inter-assay and background controls. Millipore Multiscreen HTS plates (Millipore, Danvers, MA) were soaked with PBS-T using a ELx50 plate washer (Biotek Instruments Inc., Winooski, VT) for 2 min. The solution was aspirated from the plates and 50 μl of each diluted standard serum or milk samples were applied to the plates. Then, 50 μl of bead solution was added to each well and incubated for 30 min on a shaker at room temperature. Then, the plate was washed with PBS-T, and 50 μl of biotinylated goat anti-bovine IgG (H+L) detection antibody (Jackson Immunoresearch Laboratories, West Grove, PA) diluted 1:1,000 in blocking buffer was added to each well and incubated for 30 min as above. After washing, 50 μl of streptavidin-phycoerythrin (Invitrogen, Carlsbad, CA) diluted 1:100 in blocking buffer was added. Plates were incubated for 30 min as above and washed. The beads were resuspended in 100 μl of blocking buffer and the plate was placed on the shaker for 15 min. The assay was analyzed in a Luminex 200 instrument (Luminex Corp., Austin, TX). The data were reported as median fluorescent intensities (MFIs).

### Recombinant MAP protein ELISA

Assays were conducted with serum samples from NL (n = 30) and F+E+ groups (n = 60) using 6 recombinant MAP proteins that were applied in the multiplex assays. The procedure was adapted from the previously described protocol [25] with a minor modification. ELISA 96-well microplates were coated with 50 μl/well of MBP-tagged recombinant MAP protein (1 μg/ml) or MBP/LacZ fusion protein (0.5 μg/ml) in carbonate/bicarbonate buffer [0.1 M pH 9.6]. Plates were sealed and incubated overnight at 4°C, then washed three times with 1xPBS, pH 7.4 containing 0.1% Tween 20 (PBS-T). Wells were blocked by adding 200 μl of PBS-T containing 1% bovine serum albumin (PBS-T-BSA) and incubated at room temperature for 1 hour before washing the plate three times with PBS-T. Serum samples diluted 1:250 in PBS-T-BSA were added to each well (100 μl) and incubated at room temperature for 1 hour before washing six times with PBS-T. Then anti-goat IgG peroxidase conjugate (Vector Labs, Burlingame, CA, USA) diluted 1:10,000 in PBS-T-BSA was added to all wells (100 μl) and incubated at room temperature for 1 hour before the plates were again washed six times with PBS-T. Finally, 100 μl/well of tetra methylbenzidine (TMB) SureBlue solution (KPL, Gaithersburg, MD, USA) was added and the reaction incubated for 10–15 minutes at room temperature with no light, before the reaction was stopped with 100 μl/well of 1.0 N HCl solution. The spectrophotometric reading of all wells was performed at 450 nm using a PowerWave XS2 microplate reader (BioTek, Winooski, VT, USA). The OD value of each sample was normalized by [sample OD–MBP/LacZ OD] to eliminate the background produced by the non-specific binding.

### Statistical analysis

The group comparison was conducted using one-tailed Mann-Whitney U tests with a significance level at p < 0.05 (also called the Wilcoxon Rank-Sum test) to compare MFI values in serum and milk assays in F+E- and F+E+ groups as compared to the NL (http://www.socscistatistics.com/tests/mannwhitney/). P-value adjustments were made because multiple statistical tests were performed on the same sample set (e.g. set 1 = NL vs. F+E-, set 2 = NL vs. F+E+); a Bonferroni correction was applied to alpha (0.05/(number of tests performed)). To determine the sensitivity and specificity for each antigen within the multiplex assay, a Receiver Operating Characteristic (ROC) curve was generated using the ROCR package in the R program (https://www.R-project.org/). The cutoffs for sensitivity and specificity were based on maximum Youden Index (*J* = Se + Sp -1) [26]. The agreement between serum and milk reactivity to each antigen (MFI) was analyzed with Spearman rank correlation (http://www.socscistatistics.com/tests/spearman/Default.aspx). The concordance correlation was generated using the Agreement package in R. The Strength of agreement was estimated by Covariance R and the concordance correlation coefficient (CCC) with < 0.65 as poor, 0.65–0.8 moderate, 0.8–0.9 substantial, and >0.9 almost perfect.

## Results

### Immunological and microbiological assessment of MAP infection status

The samples used in our current studies were from animals tested for MAP infection status using ELISA kits (2 for serum and 1 for milk), five fecal assays including three cultures (1 solid and 2 liquid) and two commercial qPCR assays as part of the JDIP diagnostic standards sample collection project (Table 1). All samples from cows in the NL group (from uninfected herds) were negative in each of the eight assays, while 70% of those in the F+E+ group tested positive in all 8 assays, 23.3% positive in 7, and 6.7% in at least 6 of the assays. For animals in the F+E- group, ELISA tests were negative in all cows; while 70% of animals tested positive in at least two of the three fecal culture assays, and the remaining 30% were positive for at least one. The results also showed that 60% of all cows in the F+E- group cows tested positive only with one or more qPCR assays while the remaining 40% had at least 1 positive in culture tests with or without qPCR positive. The fecal qPCR Ct values were significantly lower in the F+E+ group compared to the F+E- group (P<0.001), indicating a considerably higher level of shedding in F+E+ cows (Table 1). The number of lactations and the days in milk (DIM) were comparable in all three groups, and although the values were slightly higher for lactation number and DIM for the F+E- and F+E+ groups compared with the NL group, they were not significant (Table 1).

**Table 1:** Assessment of MAP infection status in 180 samples in this study.

Tests	Statement	NL	F+E-	F+E+
Serum ELISA (IDEXX)	Pos (%)	0.00	0.00	100.00
Serum ELISA (ParaChek)	Pos (%)	0.00	0.00	93.33
Milk ELISA (IDEXX)	Pos (%)	0.00	0.00	91.38 (Susp 3.45)
Fecal culture (HEYM)	Pos (%)	0.00	10.00	85.00
Fecal culture (MGIT)	Pos (%)	0.00	15.00	98.33
fecal culture (Trek)	Pos (%)	0.00	33.33	100.00
qPCR (LT TaqMan)	Pos (%)	0.00	85.00	100.00
qPCR (Tetracore)	Pos (%)	0.00	47.00 (Susp 23.00)	98.30 (Susp 1.70)
LT TaqMan Ct value	M±SD	> 40	35.55±2.74	26.70±4.05
P value			vs NL (<0.0001)	vs F+E- (<0.0001)
Tetracore Ct value	M±SD	> 40	37.69±2.52	29.77±4.16
P value (unpaired, 2 tails)			vs NL (<0.0001)	vs F+E- (<0.0001)
Lactation number	M±SD	2.90±1.27	2.95±1.06	3.32±1.40
P value (vs NL)			0.815	0.088
Days in Milk	M±SD	166.08±128.45	181.52±150.39	195.72±135.97
P value (vs NL)			0.547	0.222

### Serum and milk multiplex immunoassays

**Fig 1:**
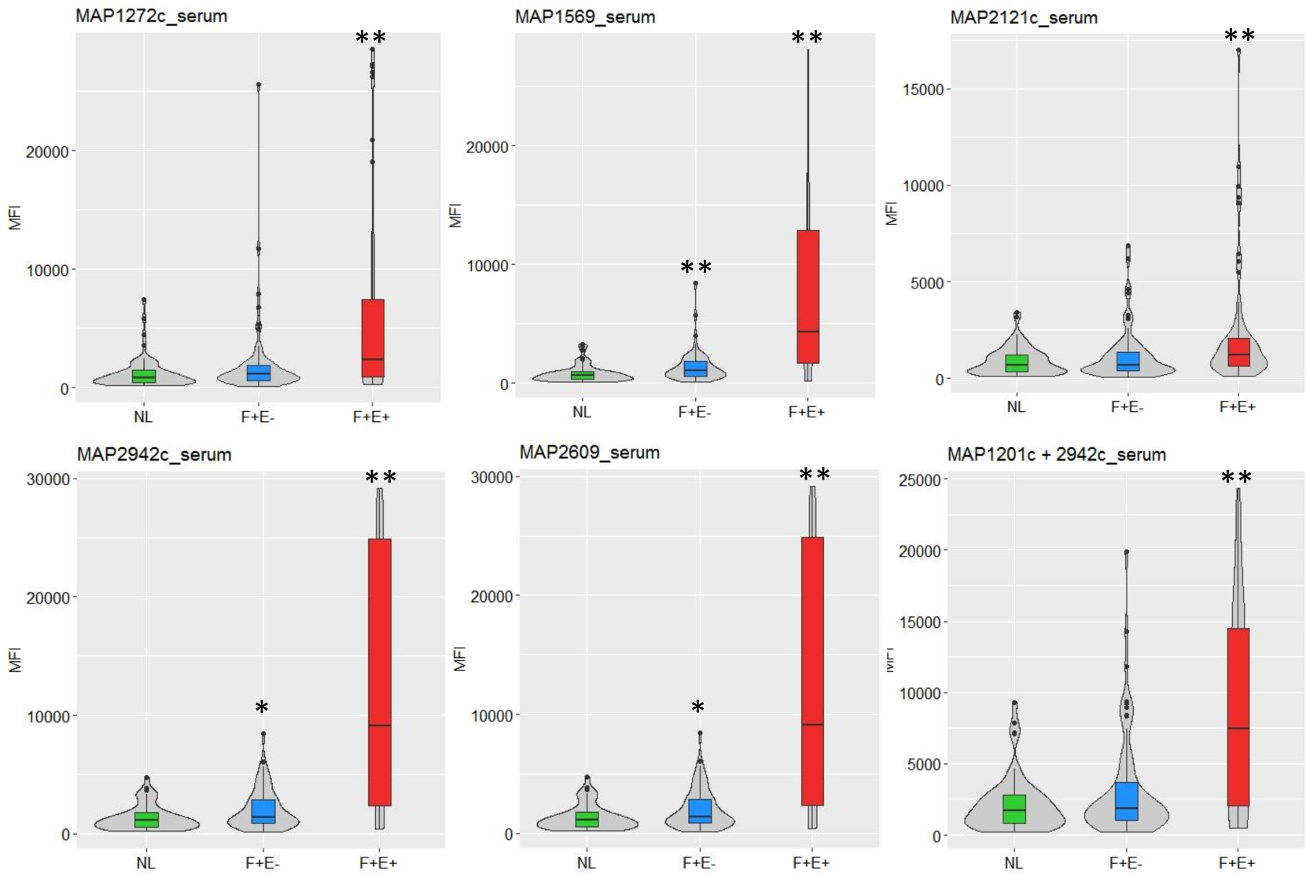
Distribution of serum multiplex assay median fluorescent intensity (MFI) to each antigen among groups. The violin plots (gray-filled) show the distribution shape of the data among NL (n = 60), F+E- (n = 60), and F+E+ (n = 60). The box plots in the center represent the interquartile range. The vertical line on each box represents 1.5x interquartile range (IQR), and the dots represent outliers. The symbol * indicates p < 0.05 when MFI in infected groups (F+E- or F+E+) compared to MFI in NL group, and ** indicates p < 0.01 based on Mann-Whitney’s U test.

**Fig 2:**
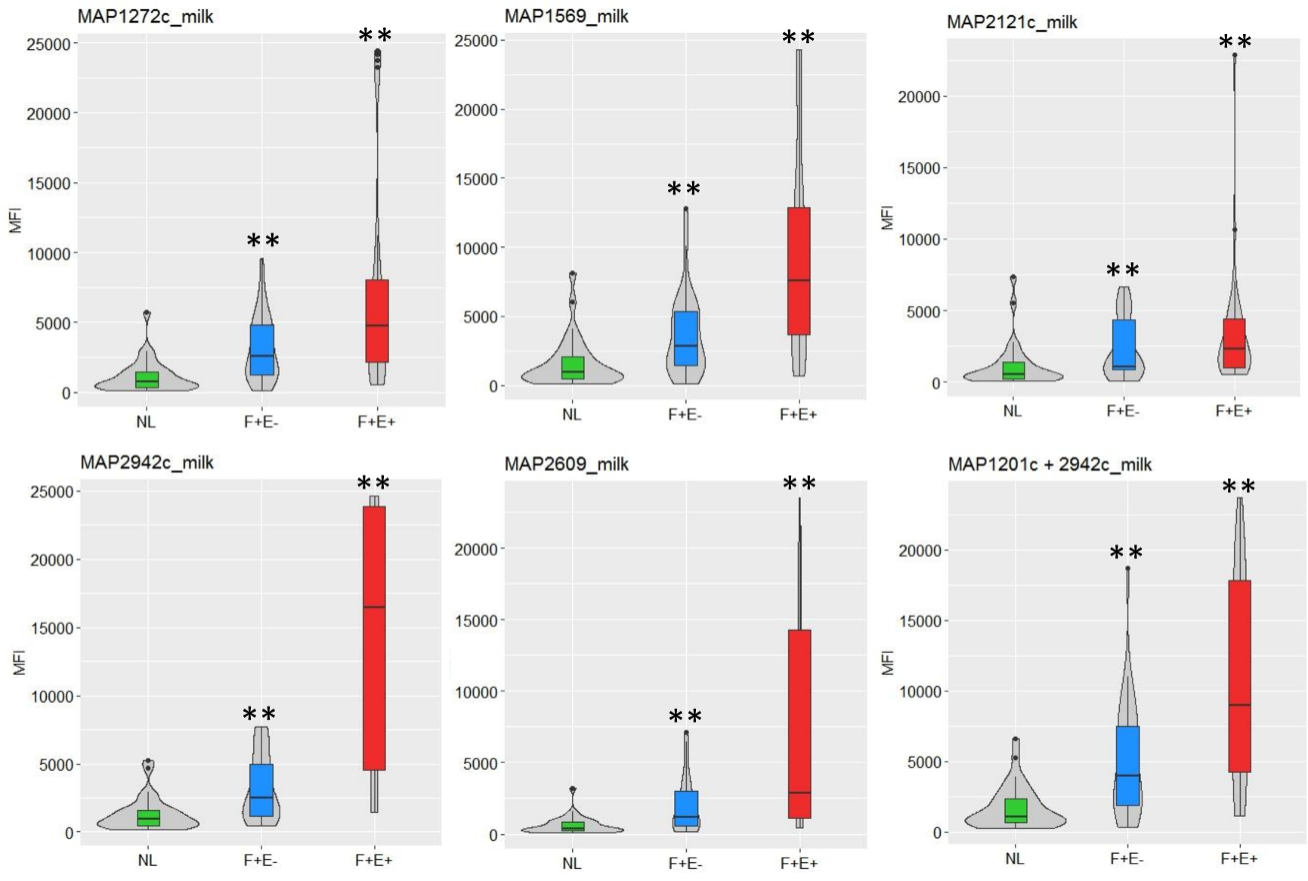
Distribution of milk multiplex assay MFI to each antigen among groups. The violin plots (gray-filled) show the distribution shape of the data among the NL (n = 30), F+E- (n = 30), and F+E+ (n = 30) groups. The box plots in the center represent the interquartile range. The vertical line on each box represents 1.5x interquartile range (IQR), and the dots represent outliers. The symbol ** indicates p < 0.01 based on Mann-Whitney’s U test when MFI in infected groups (F+E- or F+E+) compared to MFI in NL group.

Samples from animals in all three groups, NL, F+E-, and F+E+, were analyzed for all six antigens, for both serum (Fig 1) and milk (Fig 2). To assess the immunogenicity of each antigen, the MFI values of samples from animals in the infected groups (F+E+ and F+E-) were compared with those from the control group (NL). The results show that, when considering the 60 serum samples from each of the NL, F+E-, and F+E+ groups, the immunoreactivity of serum from animals in the F+E- group was significantly higher for only 3 of the antigens, MAP1569, MAP2609,and MAP2942c, when compared with the NL (Fig 1, Table 2). In contrast, immunoreactivity of serum from animals in the F+E+ group was significantly higher for all the six antigens as compared with the NL group (*p*<0.001). Interestingly, for the milk samples, the results show that the immunogenicity of all six antigens was significantly higher in both F+E- and F+E+ groups (p<0.01) as compared with the NL (Fig 2, Table 2). The ratio of average MFIs in the F+E- to that in the NL for each of the antigens ranged from 1.4 to 1.7 (median 1.6) in serum and 2.0 to 3.1 (median 2.6) in milk. The highest ratio in serum was for MAP1569 and MAP1272c, and for MAP2609 in milk; the lowest ratio in both serum and milk was MAP2121c. The median ratio for F+E+/NL was 7.3 in serum and 6.5 in milk, MAP1569, MAP2942c, and MAP2609 showed the highest (9.8–9.9) in serum while MAP2942c and MAP2609 showed the highest (11.2–11.5) in milk. Again, MAP2121c showed the lowest ratio in the F+E+ for both serum and milk (Table 2).

**Table 2:** Group comparison of serum and milk MFI values (Mann-Whitney test).

Sample Type	MAP1272c	MAP1569	MAP2121c	MAP2942c	MAP2609	MAP1201c + 2942c
**Serum, n = 180**						
NL (M±SD)	1217.8±	836.3±	891.0±	1336.1±	740.2±	2129.4±
	1327.7	705.2	723.4	1047.5	455.2	1906.1
F+E- (M±SD)	2086.2±	1410.0±	1246.9±	2086.9±	1207.2±	3257.7±
	3674.0	1353.2	1402.1	1696.0	1132.1	3762.1
F+E+ (M±SD)	5877.7±	8168.3±	2343.7±	13113.7±	7334.6±	8683.5±
	8035.5	8530.8	3133.9	10854.7	7839.0	6921.8
Ratio (F+E-/NL)	1.7	1.7	1.4	1.6	1.6	1.5
P (F+E- vs NL)	0.03005	0.00042	0.1423	0.00621	0.02275	0.11123
Ratio (F+E+/NL)	4.8	9.8	2.6	9.8	9.9	4.1
P (F+E+ vs NL)	< .00001	< .00001	0.0002	< .00001	< .00001	< .00001
**Milk, n = 90**						
NL (M±SD)	1122.5±	1633.2±	1156.6±	1320.4±	621.5±	1746.5±
	1172.3	1824.8	1619.2	1234	629.7	1538.9
F+E- (M±SD)	3168.9±	3588.6±	2350.0±	3220.1±	1900.7±	5160.0±
	2331	2915.2	2105.2	2405	1873.3	4055.4
F+E+ (M±SD)	7466.1±	9374.5±	3440.4±	14749.0±	7162.6±	10942.8±
	7998.8	7042.6	4031.8	9321.9	7564.9	7310.4
Ratio (F+E-/NL)	2.8	2.2	2.0	2.4	3.1	3.0
P (F+E- vs NL)	0.00003	0.00056	0.00154	0.00016	0.0004	< .00001
Ratio (F+E+/NL)	6.7	5.7	3.0	11.2	11.5	6.3
P (F+E+ vs NL)	< .00001	< .00001	< .00001	< .00001	< .00001	< .00001

### ROC analysis of each MAP antigen for serum and milk samples

ROC analysis for the 6 antigens was performed with the 180 serum samples and the 90 milk samples (S1 Fig) and the area under curve (AUC), preliminary sensitivity and specificity were determined for each antigen individually as well as in combination based on cutoff values at maximum Youden Index (Table 3, S1 Table). The AUCs for serum in all samples for each antigen ranged from 0.63 (MAP2121c) to 0.79 (MAP1569) with median 0.71. The AUCs for milk generally were higher than the corresponding values for serum and ranged from 0.77 (MAP2121c) to 0.87 (MAP1201c+2942c) with a median 0.828 (Table 3). We also calculated ROC curves for each of the F+E+, F+E-, and Overall (F+E+ and F+E-) groups individually (Table 3), and AUCs ranged from 0.70 (MAP2121c) to 0.90 (MAP1569) with median 0.839 in serum in the F+E+ group, and 0.81 (MAP2121c) to 0.97 (MAP2942c) in milk. As expected, these were lower for the F+E- group, ranging from 0.56 (MAP2121c) to 0.68 (MAP1569) in serum and 0.723 (MAP2121c) to 0.811 (MAP1201c+2942c) in milk.

**Table 3:** ROC analysis of MAP recombinant proteins.

Antigen	AUC			
		F+E+	F+E-	Overall (F+E+ and F+E-)
MAP1272c	Serum	0.7667	0.5994	0.6831
	Milk	0.8406	0.8011	0.8011
MAP1569	Serum	0.9001	0.6768	0.7884
	Milk	0.8944	0.7456	0.8200
MAP2121c	Serum	0.6974	0.5567	0.6270
	Milk	0.8122	0.7233	0.7678
MAP2942c	Serum	0.8911	0.6322	0.7617
	Milk	0.9656	0.7711	0.8683
MAP2609	Serum	0.8704	0.6061	0.7383
	Milk	0.9189	0.7522	0.8356
MAP1201c +	Serum	0.8083	0.5645	0.6865
2942c	Milk	0.9367	0.8111	0.8739

**Fig 3:**
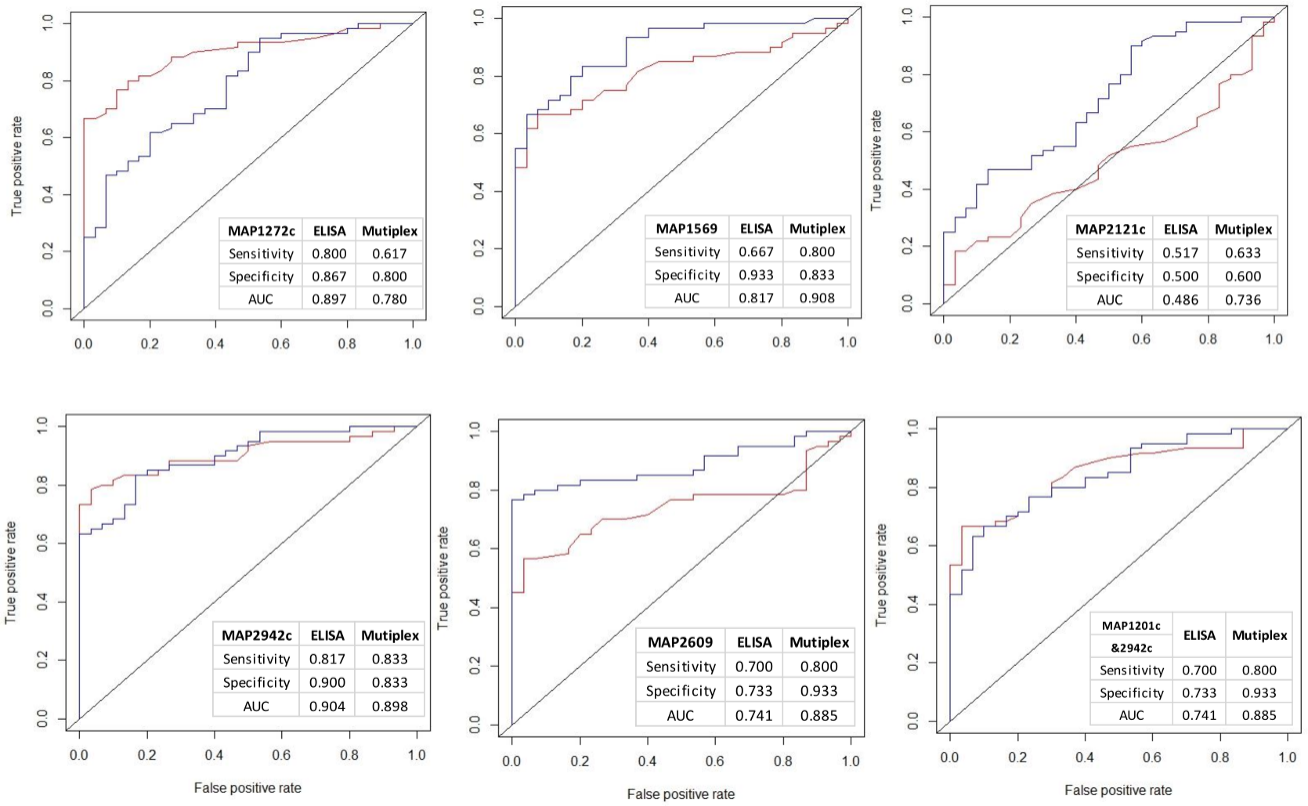
Comparison of serum antibody reactivity of multiplex and ELISA to recombinant proteins. ROC curves of serum multiplex reactivity to 6 recombinant MAP proteins were compared with those of serum ELISA using the same recombinant antigens (NL n = 30, F+E+ n = 60). The red ROC curves represent data from serum ELISA and blue ROC curves represent data from multiplex assay. The tables inside the plots describe the name of antigen, sensitivity, specificity and AUC.

**Fig 4:**
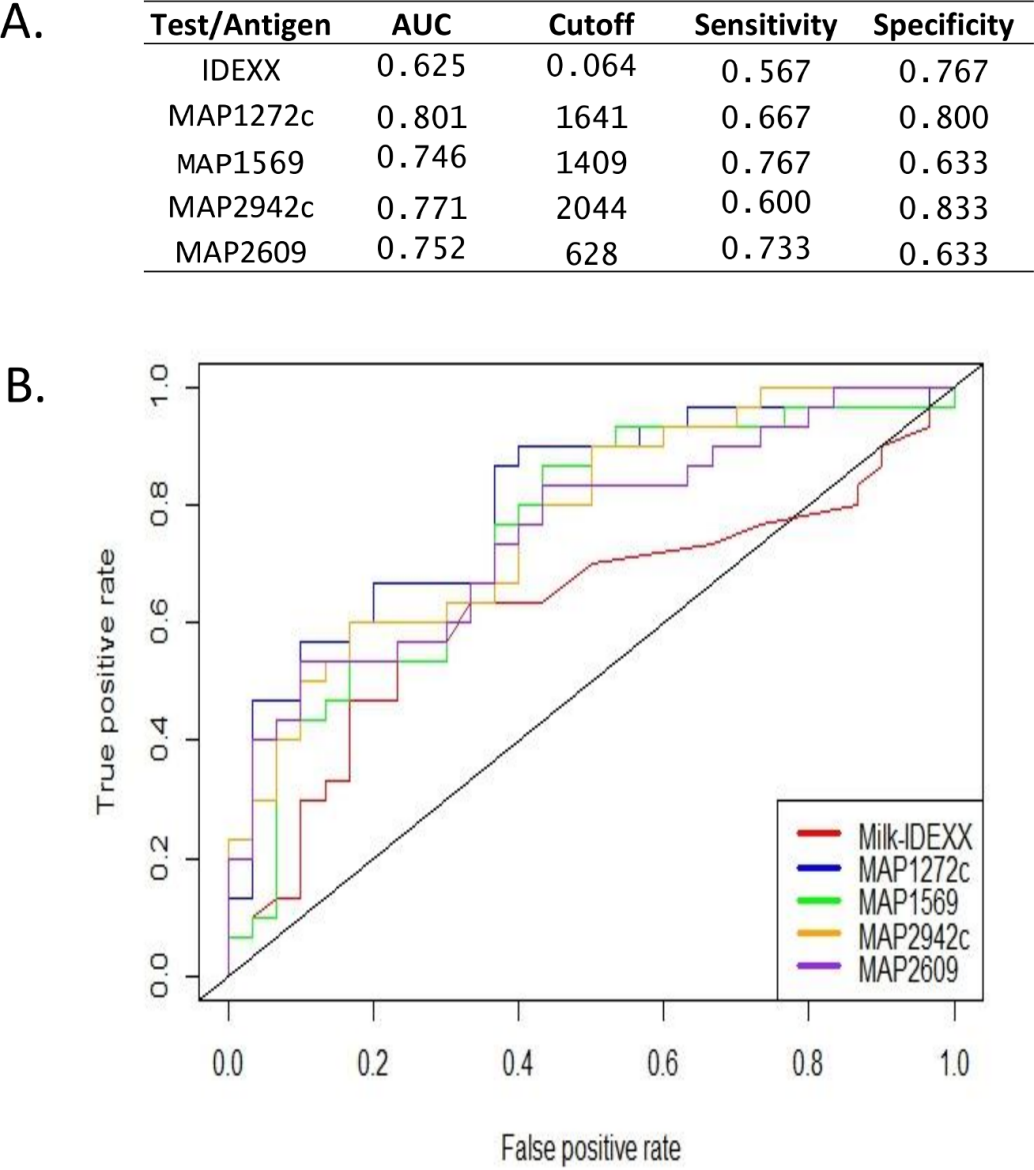
Comparison of milk multiplex and ELISA antibody reactivity. Milk multiplex antibody reactivity to recombinant MAP proteins was compared with IDEXX ELISA test results in the F+E- (n = 30) and NL (n = 30). (A) Table of AUC, cutoff, Sensitivity and Specificity; (B) ROC curves.

Next, we compared the ROC curves of serum samples generated from the multiplex assays with those from the ELISA using the same recombinant MAP antigens and noted higher multiplex AUCs in MAP1569, MAP2121c and MAP2942c and similar AUCs in the other three proteins (Fig 3). This suggests that the multiplex test has higher sensitivity and specificity compared to using the same antigens in regular ELISA tests. We also compared ROC curves of milk multiplex results with those of milk ELISA using commercial IDEXX kits in the F+E- group. Multiplex AUCs of recombinant proteins were all higher compared to that obtained using IDEXX kits (Fig 4), indicating an advantage of the multiplex assay in detection of early infection compared to commercial ELISA kits (Fig 4).

### Concordance between serum and milk assays to individual MAP antigens

The agreement of serum and milk antibody reactivity was analyzed using the Spearman rank correlation and concordance correlation. The Spearman covariance R value ranged from 0.572 (MAP2121c) to 0.756 (MAP2942c) with median 0.661 (Table 4). The correlation between serum and milk for all antigens was significant (p <0.01). The concordance correlation coefficient (CCC) ranged from a relatively poor 0.55 (MAP2121c) to a moderate 0.79 (MAP2942c) with median CCC of 0.69 (Table 4). As noted earlier, the highest levels of precision and accuracy for both serum and milk were observed for MAP2942c.

**Table 4:** Concordance correlation between MFI values from serum and milk assays.

Antigens	Spearman correlation		Concordance		
	covariance R	p value	CCC	Precision	Accuracy
MAP1272c	0.587	<0.01	0.6183	0.6475	0.9549
MAP1569	0.673	<0.01	0.6174	0.7049	0.8758
MAP2121c	0.572	<0.01	0.5529	0.6115	0.9041
MAP2942c	0.756	<0.01	0.7947	0.8104	0.9807
MAP2609	0.649	<0.01	0.6839	0.6926	0.9874
MAP1201c+2942c	0.726	<0.01	0.6971	0.7353	0.948
6 Ags combined	0.677	<0.01	0.6917	0.7207	0.9598
4 Ags combined	0.668	<0.01	0.6923	0.7172	0.9653

### Increased sensitivity by using a combination of recombinant antigens

With the caveat that these are preliminary studies with a selected group of samples that preclude robust estimates of sensitivity and specificity, we noted from the ROC curves, the sensitivity of a single antigen assay was low, especially for the F+E- group. Therefore we tested whether using a combination of antigens increases the sensitivity. With the ROC cutoff (at maximum Youden Index), we calculated the sensitivity with a combination of all 6 antigens and the 4 most reactive antigens (S1 Table). In serum samples from the F+E+ group, the assay sensitivity increased from 0.63–0.81 using single antigens to 0.95 and 0.97 with 4- and 6-combined antigens, respectively. However, the assay specificity was reduced to 0.70 and 0.53 with 4- and 6-combined antigens. The four-antigen combination increased the specificity without obvious loss of sensitivity as compared to the combination of 6 antigens. To explore alternative approaches to increase assay specificity, we applied a cut-off using the mean+2SD of the NL, and re-estimated the sensitivity and specificity each antigen individually and in combination (Table 5). This increased (for the 4-antigen combination) predicted specificities of the assay in serum and milk to 0.87 and 0.90, respectively, and the sensitivity increased to 0.90 for serum and 0.93 for milk in the F+E+ group. As expected, although higher than single antigen (0.1–0.217 in serum, 0.27–0.47 in milk), the sensitivity of the combined 4-antigen assay is still lower in the F+E- group with 0.38 in serum and 0.57 in milk.

**Table 5:** Sensitivity and Specificity for serum and milk at M+2SD cutoff.

Sample Type	MAP1272c	MAP1569	MAP2121c	MAP2942c	MAP2609	MAP1201c + 2942c	*4-combined	**6-combined
Serum (n = 180)								
Specificity	0.950	0.950	0.967	0.950	0.983	0.933	0.867	0.783
Overall Sensitivity	0.200	0.408	0.183	0.408	0.417	0.350	0.642	0.675
Sensitivity_F+E-	0.100	0.167	0.133	0.183	0.217	0.150	0.383	0.433
Sensitivity_F+E+	0.300	0.650	0.233	0.633	0.617	0.550	0.900	0.917
Milk (n = 90)								
Specificity	0.967	0.933	0.933	0.933	0.967	0.933	0.900	0.867
Overall Sensitivity	0.517	0.417	0.300	0.583	0.467	0.583	0.750	0.783
F+E- Sensitivity	0.433	0.267	0.300	0.367	0.333	0.467	0.567	0.633
F+E+ Sensitivity	0.600	0.567	0.300	0.800	0.600	0.700	0.933	0.933

*4-combined: combination of MAP1272c, MAP1569, MAP2942c, and MAP2609

**6-combined: combination of all 6 antigens

## Discussion

Fluorescent bead-based multiplex assays have been rapidly gaining popularity for use in clinical microbiology and diagnostic laboratories due to their enhanced sensitivity and greater dynamic quantification range [27]. Despite these advantages, bead-based multiplex assays have not been tested for clinical diagnostic use in Johne’s disease in animals. The results of our investigation demonstrate the feasibility of developing sensitive and specific immunoassays for the simultaneous detection of antibodies to selected MAP recombinant proteins in serum and milk samples from infected cows, especially during early infection in animals that are fecal test positive but negative with traditional commercial ELISA kits.

The results show that when used in combinations of up to 4 recombinant MAP antigens, more than 90% of infected cows in the F+E+ group were recognized (90% with serum and 93.3% with milk) with a specificity of 0.867 and 0.900. In the F+E- group in which all animals tested negative with two independent serum and one milk ELISA test kits, 38.3% and 56.7% of infected animals were successfully identified in serum and milk respectively, suggesting a higher sensitivity of the multiplex assay format for detection of cows during early stages of infection compared to all currently available ELISA tests. Importantly, with the exception of MAP1272c, the serum multiplex bead-based assays consistently showed higher sensitivity and specificity than the corresponding values for the ELISA (Fig 3). We acknowledge that the sample set in this study is small and selected and that a robust determination of sensitivity and specificity must be based on a large collection of unbiased field samples in our future studies. Nevertheless, the existing data support the advantages of recombinant MAP antigen-based multiplex testing for improving specificity and sensitivity of serological Johne’s assays and also suggest the feasibility of a multiplex Johne’s assay for identifying infection in many animal before it can be reliably detected by current commercial ELISA kits.

Commercial milk ELISAs based whole MAP antigen preparations are commonly used for diagnosis of MAP infection in dairy cows. Antibody reactivity to individual MAP proteins in milk has not been evaluated in previous studies. This study demonstrated that individual MAP proteins are recognized by antibodies in milk samples during early MAP infection. Moreover, the milk assay using the same MAP antigens showed even higher sensitivity and specificity than the respective serum assay. Compared to the NL group, elevated amounts of antibodies were seen in the F+E- group with all 6 recombinant MAP proteins (p<0.05) in milk while only 3 recombinant proteins were recognized using sera. This suggests that multiplex assays could be easily adapted to the milk sampling format, although further validation in a larger number of milk samples needs to be performed in future studies. In contrast to human milk, where IgA is the dominant antibody class, IgG is typically greater than 75% of total immunoglobulin content in cow (or goat, sheep) colostrum and milk [28,29]. Therefore, in the current multiplex immunoassays in milk, most of the reactivity can likely be associated with IgG.

Previous studies investigating factors that influence the outcome of MAP ELISA in milk have suggested the role of a number of factors including milk yield (concentration of MAP-specific antibodies, mainly related to days in milk, DIM), herd (prevalence of JD), and parity (related to number of lactation) were mainly attributed [30,31]. In our investigation, days in milk (DIM) and lactation numbers were considered for animals in each group, and the results show no significant difference for DIM and lactation number between groups (Table 1). Considering that milk from a cow is easily obtained in a non-invasive manner with lower cost compared with the collection of serum, our studies suggest that it may be feasible to develop milk-based rapid and sensitive multiplex assays for the early detection of MAP infection in dairy animals.

Of the six candidate antigens tested in the multiplex assays, three antigens (MAP1569, MAP2942c, and MAP2609) showed significantly increased MFIs on group comparison and higher AUC on their ROC curves in the F+E- group, indicating higher sensitivity for detecting antibody responses in cows with early-infection. MAP1569, a secreted protein, was also identified from MAP culture filtrates and previously shown to be recognized by sera from MAP-infected cows [32]. The recombinant MAP1569 (ModD) protein was evaluated as an antigen with serum samples from infected and control cattle (infected n = 444, control n = 412) by ELISA, and ROC analysis showed AUC 0.533 in cows that were fecal culture-positive for MAP and control negative cows [16]. This is significantly lower than the AUC 0.788 in all serum samples with multiplex assay in this study, and even lower than AUC 0.677 in the F+E- group (Table 3). Similarly, secreted proteins MAP2942c and MAP2609, were also investigated in previous studies and shown to be recognized by sera from infected cows, though only a small number of sera (*n* = 11) were tested [33]. The other 3 candidate antigens evaluated in this study (MAP1272c, MAP2121c, and MAP1201c+2942c) were not able to detect infection in the F+E- group with serum assay, but were able to detect infection in the milk assay. Although the response to MAP1272c was not significantly higher in F+E- than in the control (NL), its addition to the combination of antigens increased the sensitivity. MAP2121c in both serum and milk ROC analysis showed the lowest specificity (serum 0.583 and milk 0.667), suggesting it may not be a good candidate for use in an immunodiagnostic setting.

Curiously, the results suggest that the fusion protein MAP1201c+2942c did not exhibit increased antibody reactivity as compared with MAP2942c alone. Additionally, higher background was seen in this fusion protein compared to MAP2942c alone, suggesting that careful attention will need to be paid for reducing specificity when using fusion proteins for assays of this nature, particularly since it is relatively easy to include or exclude specific antigens to increase sensitivity or discriminatory power using the bead-based multiplex assays.

The studies show that despite the fact that the new multiplex assays are more sensitive than the existing ones in the F+E- group, the specificity and sensitivity values still need further improvement for reliable early serological diagnostic of Johne’s disease. While there are many potential biological factors that could contribute to this finding, we note that one simple explanation for the low specificity values may also be that cows that are actually exposed and infected were not recognized as such with the existing low sensitivity assays, and hence treated as “negative” when they were actually “positive”, considering several studies have previously reported that MAP was recovered from tissues of cattle during slaughter despite negative fecal culture or PCR tests and being from “low” prevalence herds [34–36]. We carefully analyzed the cows in the NL group considered as the “true negatives” in our study. These cows were all from two herds, 33 were from herd A (herd size 222) and 27 from herd G (size 287), and both herds were categorized as uninfected based on a prevalence (rate 0%) with ELISA tests one year before sample collection. Samples, including serum, milk, and feces, were collected from 136 cows in herd A and 175 cows in herd G, and examined with serum and milk ELISAs, fecal cultures, and fecal PCRs. If a cow with any one positive of the 8 tests is considered as infected, there were 10 from herd A and 5 from herd G, which indicates infected cows possibly existed in these two “uninfected” herds, and the results of the specificity and sensitivity analyses have to be considered in this light.

An additional source of non-specific reactivity may have resulted from the inclusion of MBP as part of the MAP fusion protein to facilitate proper folding and solubilization of the expressed proteins [24,37]. Since MBP has previously been shown to be recognized by sera from a small number of cattle and sheep, and antigenicity after cleavage and removal of MBP has been shown to be marginally enhanced [24,38], future studies may need to consider the inclusion of controls with beads-coupled with MBP or use recombinant proteins without the MBP tag [38] to help reduce non-specific binding.

Finally, taken together in context of the fact that the candidate proteins evaluated in this study represented only a small subset of those that were found to be immunogenic using sera from our previous MTB and MAP protein array studies [17], it is quite likely that the screening of additional recombinant MAP proteins in future studies. In particular, antigens that are able to better discriminate the F+E- group, may provide considerable potential to further enhance the sensitivity and specificity of the multiplex assay for detection of MAP infected animals during the early stages of infection and thereby help with disease control efforts.

## Supporting information

S1 Fig ROC curves depicting reactivity for each antigen with both serum and milk samples. (A) Serum (n = 180, 60 each group), (B) Milk (n = 90, 30 each group). Group All represents 180 samples in serum and 90 in milk; F+E+/NL includes group NL and F+E+ (n = 120 in serum; n = 90 in milk); F+E-/NL includes group NL and F+E+ (n = 120 in serum; n = 90 in milk).
(TIFF)

S1 Table AUC, cut-off, sensitivity, and specificity values for each of the six MAP recombinant proteins, individually and in combination in serum and milk. (XLSX)
