# Supplementary figures and images for: Early detection of Mycobacterium avium subsp. paratuberculosis infection in cattle with multiplex-bead based immunoassays
Source: PLoS One. 2017 Dec 19;12(12):e0189783. doi: 10.1371/journal.pone.0189783 (PMC5736219; doi:10.1371/journal.pone.0189783)

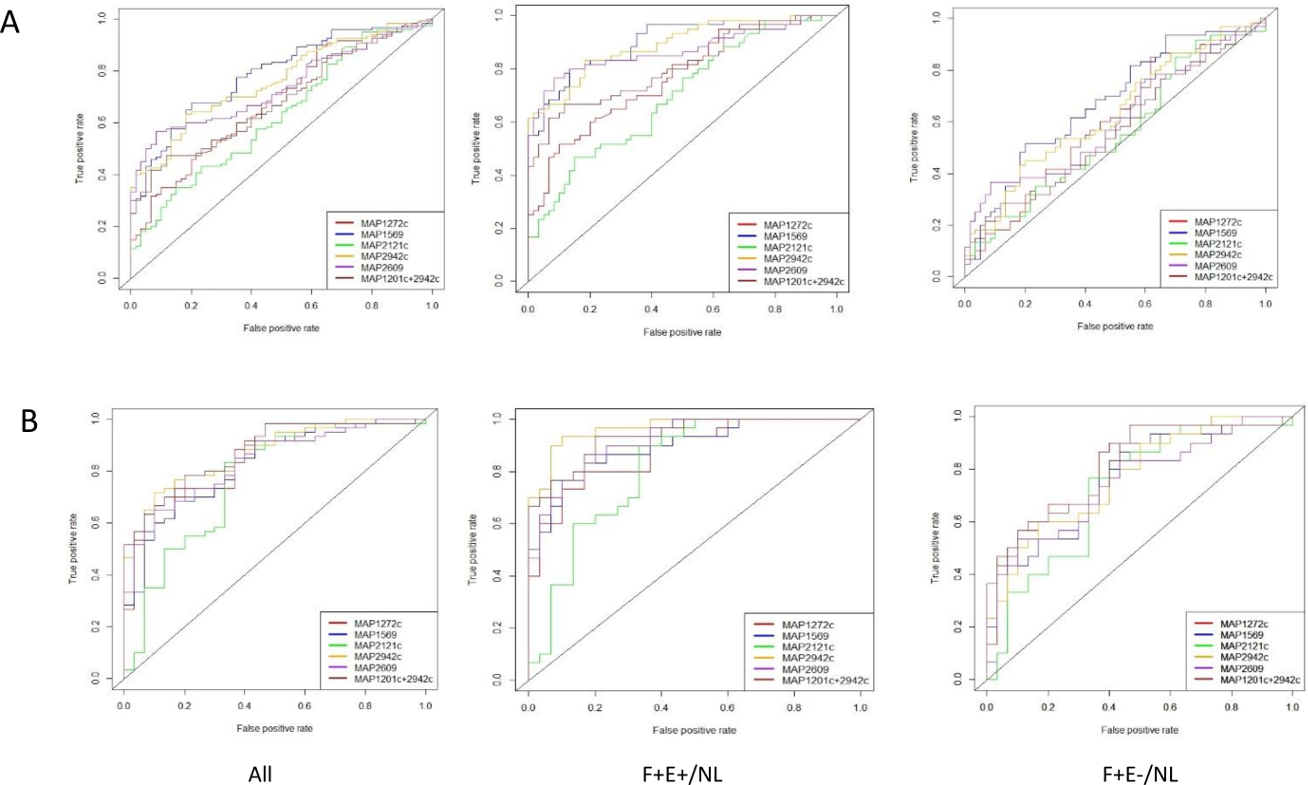

Supplement: S1 Fig — (A) Serum (n = 180, 60 each group), (B) Milk (n = 90, 30 each group). Group All represents 180 samples in serum and 90 in milk; F+E+/NL includes group NL and F+E+ (n = 120 in serum; n = 90 in milk); F+E-/NL includes group NL and F+E+ (n = 120 in serum; n = 90 in milk). (TIFF) [file pone.0189783.s001.TIFF]
